# Unisexual flowers as a resolution to intralocus sexual conflict in hermaphrodites

**DOI:** 10.1098/rspb.2023.2137

**Published:** 2023-11-29

**Authors:** Kai-Hsiu Chen, John R. Pannell

**Affiliations:** Department of Ecology and Evolution, University of Lausanne, Biophore Building, 1015 Lausanne, Switzerland

**Keywords:** sexual interference, dichogamy, sexual antagonism, sex allocation, monoecy, androdioecy

## Abstract

In dioecious populations, males and females may evolve different trait values to increase fitness through their respective sexual functions. Because hermaphrodites express both sexual functions, resolving sexual conflict is potentially more difficult for them. Here, we show that hermaphrodite plants can partially resolve sexual conflict by expressing different trait values in different male and female modules (e.g. different flowers, inflorescences, branches etc.). We analysed the flowering phenology, sex allocation and selection gradients on floral traits of flowers of the andromonoecious plant *Pulsatilla alpina*, which produces both bisexual and male flowers. Our results indicate that strong protogyny prevents early bisexual flowers from profiting from high siring opportunities early in the reproductive season at a time when male flowers could achieve high siring success. The production of unisexual male flowers thus resolves this sexual conflict because it allows the flowers to express their male function without waiting until after the female function has been performed. Our study illustrates the resolution of sexual conflict arising from phenological constraints via modular divergence in sex allocation. We discuss the extent to which modular variation in sex allocation in the context of other sexual systems may be similarly explained.

## 1. Introduction

**Figure 1.**
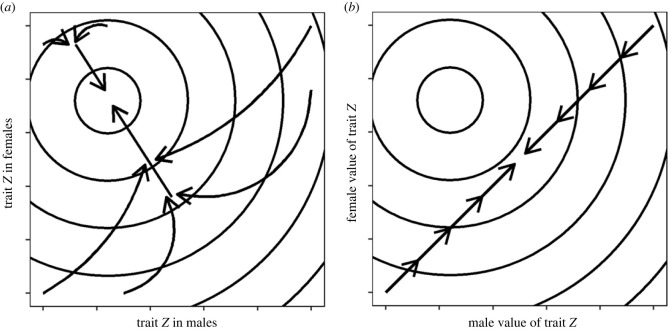
Theoretical conception of trait evolution in (*a*) dioecious and (*b*) hermaphroditic populations, as elaborated by Morgan [6]. Circles represent lines of equal total fitness for given combinations of the male and female values of trait *z*, with the smallest circle indicating the point of maximum fitness. Populations are envisaged to evolve along the arrowed lines. In (*a*), the separation of the two sexes into male and female individuals in a dioecious population allows populations to evolve towards the trait optimum for both sexual functions. In (*b*), the fact that the same individuals must express both a male and female function in a hermaphroditic population means that the fitness optimum is unreachable for traits measured at the individual level. In the text, we argue that the modularity of hermaphroditic organisms such as plants allows them to produce different male and female modules, each expressing a different (potentially optimum) value of trait *z*. Figure panels are redrawn from Morgan [6].

Intralocus sexual conflict arises when a particular trait has different optima for male and female functions. In dioecious organisms, the conflict can be (partially) resolved if the trait's value is able to diverge between males and females through the evolution of sexual dimorphism, either as a result of sex-differential gene expression [1–3] or via gene sequence divergence at loci linked to the sex-determining locus on a sex chromosome [4,5]. Persistence of conflict in such populations may then be attributed to the failure of male and female phenotypes to express their fitness optima accurately, e.g. because of a persisting correlation in a trait's expression between the two separate sexes (figure 1*a*). This same theory also applies to hermaphrodites, though with important differences [7–9]. In hermaphroditic populations, because the same individuals express both male and female functions, a trait's value at the individual level is necessarily identical for the two sexual functions, so that the male–female trait correlation must equal one and cannot be broken [6]. As a consequence, the expression of a trait by hermaphrodites will tend to be constrained to the diagonal in a plot of the trait expressed relative to the male and female functions (figure 1*b*) [6]. In this sense, the resolution of sexual conflict in hermaphrodites would appear to be more difficult than in dioecious organisms.

The idea that the male and female components of fitness of hermaphrodites may be promoted by different trait values has been much discussed in the context of the reproductive strategies of flowering plants, often in the context of mechanisms to promote outcrossing or to avoid self-fertilization and inbreeding depression (reviewed in [10]). For example, a large floral display may be beneficial to a hermaphrodite's male function because of its greater attractiveness to pollinators [11], but large floral displays can also lead to pollen transfer between flowers (geitonogamy), compromising female fitness as a result of self-fertilization and inbreeding depression in progeny [12–14]. Floral display size thus provides an example of potential intra-locus conflict between alleles that increase floral display size, thereby promoting male fitness, and alleles that reduce display size, thereby limiting self-pollination via geitonogamy and promoting the female fitness component. The floral size adopted by self-compatible hermaphroditic plants must therefore often be the result of a compromise found by natural selection between different trait values that would be optimal for each of the two sexual functions. Accordingly, many of the striking adaptations of flowers and inflorescences can be seen as ways to minimize intra-locus sexual conflict [15]. At the flower level, sexual interference, gamete wastage and reduced fitness due to the presence of the opposite sex function [15] could all be viewed as sexual conflicts on traits of sex allocation within bisexual flowers (e.g. pistil number and stamen number). For instance, the optimal functioning of male and female floral parts within bisexual flowers can be prevented by physical interference, e.g. large pistils may benefit a plant's female function, but their presence in flowers may compromise effective contact between anthers and pollinators and thereby reduce pollen export [16].

Protandry and protogyny represent particularly interesting sources of sexual conflict in hermaphrodites because of the way in which mating opportunities change during the course of a reproductive season [17]. In protogynous species with bisexual flowers, for instance, in which the female function precedes the male function, mating opportunities for individuals expressing their male function will tend to be high early in the flowering season when most of the population's flowers are in their female stage. Because protogyny means that there will be few individuals still expressing their female function towards the end of the season, mating opportunities for the male function should decline. The reverse pattern of change in mating opportunities will be manifested in protandrous species. We might therefore expect selection to favour an early onset of male function in protogynous species and its late onset in protandrous species, i.e. to favour a reversal in the temporal order of the two sexual functions [17,18]. Polymorphisms in which protandrous and protogynous individuals coexist (e.g. ‘heterodichogamy’ and ‘flexistyly’; [19–21]) represent striking examples of a resolution of this type of sexual conflict, with all individuals able to mate as male or female at times corresponding to high mate availability for the respective sexual function. Such polymorphisms break the constraint depicted in figure 1*b*. Although all individuals may be hermaphroditic in their sex allocation, they differ in when their two sexual functions are deployed. In these cases, negative frequency-dependent selection is expected to maintain the two strategies as an evolutionarily protected polymorphism.

The theoretical constraint depicted by the diagonal in figure 1*b* may also be broken by hermaphrodites that express their different sexual functions in different growth modules, e.g. in different flowers, inflorescences or ramets. Although hermaphrodite individuals might not differ in their mean trait values over all their modules (at the individual level, they are thus constrained to occupy the diagonal), the possibility of within-individual variation among modules allows them to deviate from the diagonal *at the level of their modules*. For instance, at least 7% of functionally hermaphroditic plants are monoecious [22], with separate male and female flowers that may differ markedly in size, morphology and position on the plant [23–25]. These differences between male and female floral modules presumably allow monoecious plants to optimize their two sexual functions in ways that would not be possible for plants with only bisexual flowers. In this sense, the modularity of monoecious plants allows them to resolve some elements of conflict that compromise the fitness of hermaphrodites with bisexual flowers (by producing separate male and female modules) while continuing to adopt an intermediate (hermaphroditic) sex allocation at the individual level.

The extent to which the production of unisexual flowers allows hermaphrodite plants to resolve elements of their sexual conflict could be addressed in a number of ways. One profitable approach would be to draw fitness comparisons between hermaphroditic species with bisexual and those with unisexual flowers (e.g. [26]). Another approach, which we adopt here, is to compare the fitness implications of unisexual and bisexual flowers with varying sex allocation on the same individuals. Specifically, consider the extent to which the andromonecious strategy (the production of both male and bisexual flowers) of individuals of the perennial herb *Pulsatilla alpina* (Ranunculaceae) resolves sexual conflict associated with shifting mating opportunities over their reproductive season. Because bisexual flowers of *P. alpina* are strongly protogynous, the consistent production of only bisexual flowers would prevent individuals from expressing their male function at the beginning of the reproductive season. By contrast, the suppression of the female function in some early flowers would allow them to take advantage of the high mating opportunities early in the reproductive season when most bisexual flowers are in their female phase. The flowers of *P. alpina* vary substantially in their sex allocation, but they do so more in terms of pistil number than stamen number. Because we wished to assess the fitness implications of a wide range of allocation to both male and female functions, we thus included in our study an assessment of the siring success of individuals with experimentally reduced numbers of stamens, too.

We first evaluated the extent to which the expression of the female function of bisexual flowers delays the onset of an individual's male function, and we assessed the missed mating opportunities that such delays would entail in a population in which all individuals only produce bisexual flowers. We then used paternity analysis based on genetic fingerprinting to measure the floral component of phenotypic selection on different floral traits via female and male functions within bisexual flowers to determine the degree to which selection is sexually antagonistic as a result of the mechanisms discussed above. Finally, we determined the extent to which the production of early unisexual male flowers resolves the hypothesized conflicts, i.e. by expressing trait values that more closely reflect the male fitness optimum than that of bisexual flowers. Our results suggest that andromonoecy may indeed resolve sexual conflicts in hermaphrodites within a reproductive season. They also suggest an explanation for the expression of variation in sex allocation among seasons in the form of ‘gender diphasy’, in which individuals alternate between male and hermaphroditic phases or reproduction between different reproductive seasons.

To address our aims, we assessed the strength and direction of selection on traits at the level of flowers rather than of individuals. This focus on flowers (a modular perspective) is necessary for the analysis of a heteromorphism such as andromonoecy, which by definition requires a comparison of the fitness implications of the production of male versus bisexual flowers. A key step in our approach was to apply selection gradient analysis [27] to the male and female components of fitness contributed by bisexual flowers. Rather than referring to individual fitness, therefore, we refer to ‘contributions to fitness'. Our approach has the same limitations as any study based on selection gradient analysis of components of fitness, e.g. studies measuring only female reproductive success [28–30] or fitness components of polycarpic perennials measured in only one or few reproductive seasons [31–33]. However, despite these limitations, such studies provide useful insights into how selection might be operating at different times or on different modules of growth within individuals.

## 2. Material and methods

### (a) Study species and study site

*Pulsatilla alpina* (L.) Delarbre (Ranunculaceae) is a perennial herb growing in sub-alpine to alpine habitats in central Europe [34]. Plants persist through winter as rhizomes (underground stems). Immediately after the snowmelt in spring (early May to July), several vegetative and/or reproductive shoots emerge from the ground. Depending on their size and resource status, individuals produce from zero to approximately 20 flowers, each on its own reproductive shoot [35]. Flowers may be either male or bisexual. Phenotypically male flowers bear only stamens, whereas bisexual flowers bear stamens and one to a few hundred separate pistils, each with a single ovule. Pistils mature into achenes, which we here refer to as seeds. Bisexual flowers are strongly protogynous. Both male and bisexual flowers are predominantly visited by flies, including houseflies and syrphid flies [36].

We evaluated phenotypic selection via female and male functions on five floral traits in bisexual flowers during the flowering season of 2022 in one population of *P. alpina* in the pre-Alps of Canton Vaud, Switzerland (Population S1+; 46°17′42″ N 7°09′09″ E, 1758 m.a.s.l.). The population, located on an open slope of sub-alpine grassland and covering an area of approximately 20 × 20 m, comprised approximately 150 mainly small and probably young individuals, each typically producing only a single (male or bisexual) flower during the season (electronic supplementary material, table S1). We set up a 10 × 15 m plot within the population, enclosing 135 flowering individuals using a temporally fence. We removed all floral buds from plants outside the plot at the beginning of the flowering season so that all mating was restricted to individuals within the plot.

### (b) Flowering phenology and floral traits

We labelled each flower in the plot with a paper tag. Every 3 to 4 days throughout the flowering season, from late May to late June 2022, we recorded the flowering state of each flower on a scale of 1 to 5 (male flowers) or 1 to 7 (bisexual flowers) (see table S2 in electronic supplementary material for detailed descriptions). A flower's ‘flowering date’ was labelled as the date of its opening. For each flower, we also measured the length of its largest tepal (sepals and petals are not distinguishable in the species) and the height of its stalk, as described in [36]. At the end of the flowering season, all flowers with developing fruits were enclosed in paper bags, and all their seeds were subsequently collected.

### (c) Manipulation and estimation of floral sex allocation

Flowers of *P. alpina* vary greatly in the number of their pistils and/or stamens. To determine the effect of a flower's sex allocation on its contribution to male and female fitness components, we further amplified this variation by removing, prior to dehiscence, 50% of the stamens from 16% (*N* = 19 and 9 for bisexual and male flowers, respectively) and 100% stamens from a further 9.6% (*N* = 17 bisexual flowers only) of all the flowers in the population (with flowers assigned to each treatment randomly). Experimental stamen removal amounted to an approximately 17% reduction in the total number of stamens in the population but did not change the flowering duration of manipulated flowers (see table S1 and figure S1 in electronic supplementary material for details). We photographed all flowers individually and counted the number of their pistils and stamens from the images. Stamen counts from photographs were calibrated with reference to the number of stamens counted for 15 flowers *in vivo* [36].

### (d) Estimates of pistil availability for mating

We inferred the prospective siring opportunities for stamens at each time point in our sampling by dividing the total number of receptive pistils in the population at each observation time by the total number of stamens mature at that time, for three different scenarios (see table S2 in electronic supplementary material for details): (1) based on calculations of pistil availability per stamen in the actual experimental population (i.e. as a result of the experimental stamen removal); (2) based on calculations of pistil availability per stamen produced by flowers before their removal; and (3) based on calculations of pistil availability per stamen as for scenario (2), but excluding all unisexual male flowers from the set of potential sires. The comparison of scenario (3) with either of the former two scenarios allowed us to assess the extent to which the production of only bisexual flowers gave rise to sexual conflict, and the extent to which the production of male flowers resolves the potential conflict. When the female or male stage of a flower spanned more than one time point, we assumed that the flower presented an equal number of pistils or stamens, respectively, at each time point.

### (e) Estimates of male contributions to fitness and of the selfing rate

To estimate the seasonal contribution to fitness via a flower's male function, we assigned paternity to up to 10 mature seeds per family based on variation at 10 microsatellite markers (electronic supplementary material, Methods S1 and table S3). Paternity was assigned to candidate fathers using Cervus v. 3.0.7, assuming a confidence level of 80% and an error rate of 0.018. To further improve the paternity assignment, flowers whose flowering time did not overlap with the focal bisexual flower were excluded from the list of potential fathers for each of the seed families genotyped. For each seed family, we multiplied the total number of its seeds in the flower by the proportion of seeds in our sample of that flower that had been sired by a particular individual [37]. We then assigned this product to the siring success of the given sire. We estimated the selfing rate for each bisexual flower as the fraction of self-fertilized seeds in the sample by the sample size for the family.

### (f) Estimates of female contributions to fitness

We estimated the female contribution to fitness of bisexual flowers by multiplying the number of their seeds with the estimate of their selfing rates based on an inference of their paternity using microsatellites (see above) and one minus the inbreeding depression. In a parallel study (unpublished data), we estimated inbreeding depression to be 0.93, based on a comparison between the population inbreeding coefficient *F*_IS_ of the parents and progenies and on the population selfing rate, following Ritland [38].

### (g) Statistical analysis

We conducted all analyses within the R statistical framework v. 4.0.3 [39]. We used a linear mixed model (*lmer* function of *lme4* package in R; Bates *et al*. [40]) to evaluate the effect of pistil number on the delayed onset of the male function of bisexual flowers, defined as the difference between the date the flower opened and the date its anthers opened (we included only bisexual flowers whose stamens were not manipulated in this analysis). We included the date of flower opening as a random effect to account for the effects of abiotic factors such as temperature and precipitation that might have affected the phenology. The assumptions of the linear mixed model were checked using the *DHARMa* package in R [41].

A multivariate generalized least-square model (*gls* function in *nlme* package in R; [42]) was used to evaluate sexually antagonistic selection in bisexual flowers as a function of pistil number, stamen number, flowering date, tepal length and floral stalk height, following Lande & Arnold [27]. In this analysis, we included only individuals with a single bisexual flower and measurements of the five traits in order to assign female and male contributions of fitness to individual floral phenotypes. For the analysis, floral traits were standardized to a mean of zero and a standard deviation of one. Linear and quadratic terms of the standardized floral traits were set as fixed effects, with a potential interaction with sexual function evaluated to test for sexually antagonistic selection on each floral trait, i.e. when the selection gradients on the floral trait differed between female and male functions. Variance in contributions to fitness was allowed to differ between male and female functions in the *gls* model. Individual identity was set as a random effect to account for the non-independence of female and male contributions to fitness by the same bisexual flower (electronic supplementary material, Methods S2 for details of the model). We used the *emtrends* function in the *emmeans* package in R to extract the linear and quadratic estimates and their standard errors, with *p*-values calculated from the fitted model [43]. When one or both linear and quadratic estimates were significant, we inferred that the relevant trait was under directional, stabilizing or disruptive selection when there was one maximum and one minimum, one maximum and two minima, or two maxima and two minima within the phenotypic range, respectively.

We used two further indicators to evaluate sexually antagonistic selection. First, the index SAS was defined as the absolute difference in selection gradients between the two sexual functions [44,45]. Thus, the greater the difference between the gradients, the stronger the sexually antagonistic selection. However, because SAS is always positive (precluding discrimination between concordant and antagonistic selection), we also used the index SA defined by Innocenti & Morrow [46], as a complementary indicator of sexually antagonistic selection,

$$\mathrm{SA}=\frac{G_{F}G_{M}}{\sqrt{(G_{F}^{2}+G_{M}^{2})/2}},$$

where *G*_F_ and *G*_M_ are the selection gradients via female and male functions, respectively. Although SAS and SA are usually applied to quantify sexually antagonistic selection in directional selection gradients, we extended their application to quadratic selection gradients in this study.

We used a multivariate linear regression model (*lm* function in R) to evaluate the contribution to fitness by bisexual flowers as a function of the same five floral traits described above, with a similar model structure to the previous *gls* model, again following Lande & Arnold [27] (electronic supplementary material, see Methods S2 for details of the model). The sum of contributions to fitness via the two sexual functions of each flower was standardized by dividing by the mean for the respective sexual function over all individuals considered. Linear and quadratic terms of each trait were set as the fixed effects. For all quadratic gradients, we doubled the regression coefficient to obtain the correct estimate of stabilizing or disruptive selection [47].

## 3. Results

### (a) Phenology of male and female function over the course of the flowering season

**Figure 2.**
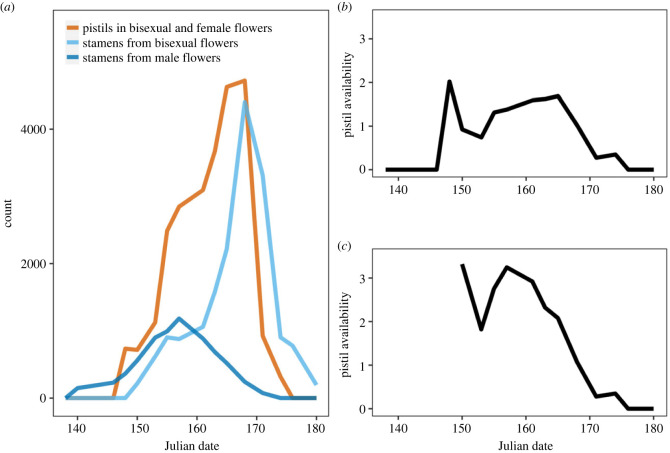
(*a*) Number of pistils in bisexual and female flowers (red line) and functional stamens from bisexual flowers (light blue line) and male flowers (dark blue line) over the course of the flowering season in the study population. (*b*) Pistil availability per stamen calculated for the study population and accounting for all bisexual and male flowers observed. (*c*) Pistil availability per stamen calculated for the study population in which only bisexual flowers are accounted for. In *b* and *c*, pistil availability was calculated by dividing the total prospective number of available pistils by an estimate of the total number of functional stamens produced by both bisexual and male flowers (*b*) or by only bisexual flowers (*c*) within each time window.

**Figure 3.**
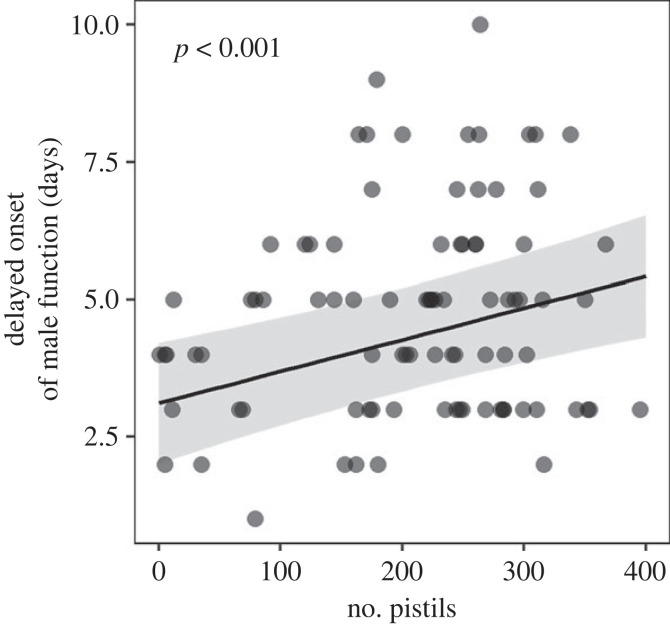
The delay in the onset of the male function of bisexual flowers as a function of the number of their pistils. The delayed onset of male function was quantified by the difference between the day a flower first opened and the beginning of its male function. Shown is the least-squares regression (line) with the 95% confidence interval (shaded).

Stamen removal manipulations had no effect on the flowering schedule of hermaphrodite flowers (electronic supplementary material, figure S1). On average, male and bisexual flowers lasted for 10.7 and 9.6 days, respectively (*t*-test, *p* < 0.01), with bisexual flowers remaining in their female state for 5 ± 2.1 days (mean ± s.d.). Unisexual male flowers were the first to begin releasing pollen in the population (dark blue line in figure 2*a*). In bisexual flowers, pistil presentation strongly preceded pollen release from stamens (compare the red and light blue lines in figure 2*a*), so their male function was strongly delayed compared with that of male flowers (see also figure S2 in electronic supplementary material). Note that the delay in the onset of the male function of bisexual flowers was not only qualitative but also depended positively on the number of their pistils (estimate: 0.58 ± 0.14 days delayed per 100 pistils; *p* < 0.001; figure 3).

The relative timing of pistil presentation and pollen release from stamens determined the temporal pattern of pistil availability per stamen over the course of the flowering season (figure 2*b*). Ignoring stamens in male flowers from the calculation of pistil availability per stamen (figure 2*c*) reveals the extent to which male flowers are able to compete for mating with pistils in the early phase of flowering, during which pistil availability is high and bisexual flowers have not yet begun to release pollen.

### (b) Sexual conflict in bisexual flowers and its resolution

A total of 892 seeds that could be genotyped for at least five loci were used for paternity analysis. We were able to assign paternity for 854 seeds to a single most likely father under a relaxed confidence interval (80%), corresponding to a 96% successful assignment rate. The male component of fitness was estimated on the basis of these assignments, while the female component of fitness was estimated on the basis of the counts of seeds produced, discounted by the product of the inferred selfing rate from the paternity analysis and the known level of inbreeding depression (0.93).

**Figure 4.**
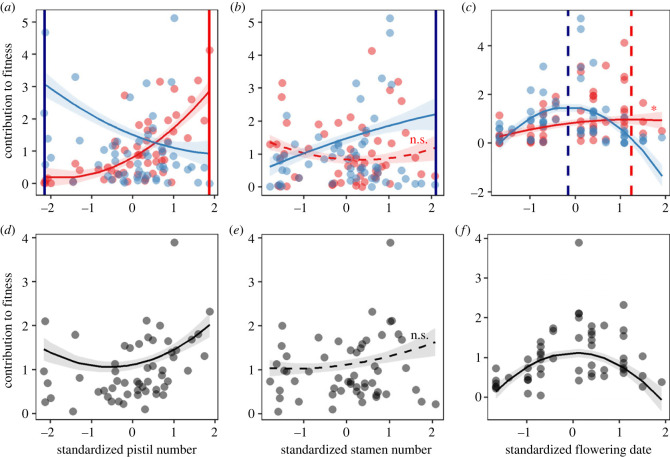
Selection gradients for contributions to fitness made through female function (red) and male function (blue) (*a*–*c*), and through both male and female functions together (grey) (*d*–*f*) as a function of pistil number (*a*,*d*), stamen number (*b*,*e*) and flowering date (*c*,*f*) in bisexual flowers. Two points are displayed for each bisexual flower in *a*, *b* and *c* (*N* = 57), one for female and one for male function. Table 1 for statistical details. Dashed regression lines indicate that neither linear nor quadratic gradients were significant. In *c*, the linear and quadratic gradients via the female function were marginally non-significant and non-significant, respectively. The shaded ribbons indicate 1 s.e. of the corresponding regression line. In *a*–*c*, optimal trait values of female and male reproductive success are indicated by red and blue vertical lines, respectively (solid lines: inference for directional selection; dashed lines: inference for stabilizing selection). For *d*–*f*, see electronic supplementary material, table S4 for the values of the linear and quadratic selection gradients.

There were clear differences in the intensity and/or direction of selection between the two sexual functions, indicating potential sexually antagonistic selection. The linear and quadratic selection gradients differed between the two sexual functions for contributions to fitness as a function of pistil number (linear gradient interaction: *p* < 0.001), stamen number (linear gradient interaction: *p* < 0.01), and flowering date (linear and quadratic gradient interactions: *p* < 0.05 and *p* < 0.01, respectively; table 1 and figure 4). The contributions to fitness through female function increased with the pistil number of bisexual flowers, but increasing pistil number decreased the contribution made by flowers through their male function. Contributions to the male component of fitness increased with the number of a flower's stamens. The flowering date was mostly under stabilizing selection for both sexual functions, especially the male function. No selection was detected on tepal length and floral stalk height (table 1).

**Table 1.** Linear and quadratic selection gradients on five floral traits of bisexual flowers in female and male functions estimated by multiple regression. Reproductive success of female and male function were estimated by the number of mature seeds produced and sired, respectively. SAS: the absolute difference in selection differentials and gradients. SA index: the concordance and strength of sexual antagonistic selection. .*p* < 0.1, *p < 0.05, **p < 0.01, ***p < 0.001.

	selection gradient	female function (s.e.)	male function (s.e.)	interaction	SAS	SA Index
pistil number	*β*	0.72 (0.15)***	−0.51 (0.19)**	***	1.22	−0.58
	*γ*_ii_	0.41 (0.19)*	0.21 (0.24)		0.2	0.26
stamen number	*β*	−0.08 (0.11)	0.42 (0.15)**	**	0.48	−0.06
	*γ*_ii_	0.24 (0.2)	−0.07 (0.26)		0.3	−0.09
flowering date	*β*	0.21 (0.12).	−0.21 (0.16)	*	0.34	−0.14
	*γ*_ii_	−0.17 (0.25)	−1.32 (0.31)***	**	1.15	0.23
tepal length	*β*	−0.04 (0.12)	0.06 (0.15)		0.09	−0.05
	*γ*_ii_	−0.07 (0.12)	0.11 (0.15)		0.18	−0.08
stalk height	*β*	−0.07 (0.14)	0.27 (0.18)		0.32	−0.06
	*γ*_ii_	−0.05 (0.19)	0.03 (0.24)		0.08	−0.04

SAS values, reflecting the absolute differences in selection gradients between the two sexes, varied from 0.08 (quadratic gradients on stalk height) to 1.22 (linear gradients on pistil number; table 1). Strongly negative values of SA, indicating strong antagonistic selection, were found for the linear gradients on pistil number (SA index = −0.58) and flowering date (SA index = −0.14; table 1).

Net selection gradients via the two sex functions were found to be disruptive and stabilizing on pistil number and flowering date, respectively (figure 4*d*,*f*; electronic supplementary material, table S3). No net selection was detected on stamen number, tepal length and stalk height (figure 4*e*; electronic supplementary material, table S4).

**Figure 5.**
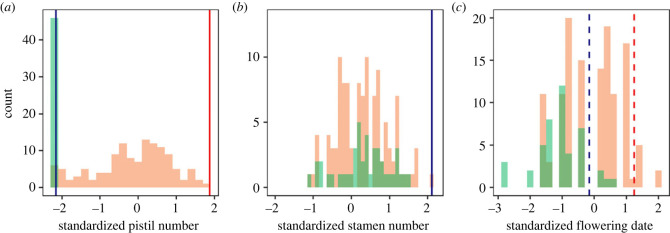
Resolution of sexual conflict via the production of male-only flowers. Histograms showing the distribution of (*a*) the number of pistils, (*b*) the number of stamens and (*c*) the flowering date for bisexual (*N* = 129, light orange bars) and male flowers (*N* = 46, light green bars) in the population before experimental manipulation of stamen number. The overlaps of the two bars are shown in dark green. Inferred optimal trait values for female and male contributions to fitness by bisexual flowers under directional and stabilizing selection are indicated, as inferred from figure 4. Note that no significant selection was detected on stamen number (*b*) for female function. Traits are standardized to the scales used in the selection gradient analysis.

Figure 5 shows the distribution of standardized pistil number, stamen number (before the manipulations), flowering date of male and bisexual flowers and the corresponding fitness optima (electronic supplementary material, table S3). For traits under sexually antagonistic selection, unisexual male flowers expressed trait values that approach the indicated optima of the male function measured in bisexual flowers (figure 5*a,c*). Male flowers flowered earlier than intact bisexual flowers (*p* < 0.001) but produced the same number of stamens (*p* = 0.8; electronic supplementary material, table S3). Comparisons of reproductive success between bisexual and male flowers are given in electronic supplementary material, figure S3.

## 4. Discussion

### (a) Protogyny leads to sexual conflicts within bisexual flowers

Our results confirm that bisexual flowers of *Pulsatilla alpina* are strongly protogynous, with their male function delayed until after their pistils have been exposed to pollinators for a few days. Given that *P. alpina* is self-compatible, this strategy of protogyny probably reduces within-flower self-fertilization, an important element of sexual interference that can compromise the female function of plants that also have a male function and in which inbreeding depression is substantial (as it is in *P. alpina*). However, if all flowers in a population are protogynous, plants producing bisexual flowers cannot capitalize through their male function on the high mate (pistil) availability early in the reproductive season. Thus, whereas protogyny may represent a resolution to one element of sexual conflict for plants with bisexual flowers (preventing self-fertilization), it creates another conflict by delaying the onset of their male function at a time when mate availability and prospective siring success are high.

The conflict affecting sex allocation in flowers of *P. alpina* arises because, on the one hand, flowers with more pistils contribute more to the female component of fitness, whereas, on the other hand, increased pistil production reduces prospects for male siring success precisely when mate availability is high. Indeed, while our selection gradient analysis revealed a negative directional selection gradient on pistil number via the male function, there was a positive directional selection gradient on pistil number via the female function, with flowers with more pistils delaying their male function longer than those with fewer pistils. The delayed onset of one sexual function is common in dichogamous species with bisexual flowers (i.e. species that are protogynous or protandrous) that prolong the first of their sexual functions. For example, in several protandrous species, the onset of the female function is determined plastically by the amount of pollen remaining within bisexual flowers [48–50] or is affected by a negative genetic correlation with the duration of the male function [51].

The conflict arising from protogyny in bisexual flowers in *P. alpina* concerns not only their sex allocation (the numbers of their pistils and stamens), but also their phenology. Our analysis indicates that selection on flowering date via male function favours early flowering, yet it favours late flowering via female function. More specifically, selection via male function was strongly stabilizing, with an optimum that was slightly earlier than the mean flowering date, whereas selection via female function mostly favoured late flowering. The inferred advantage for male function of early flowering accords with the general expectation for protogynous species in which opportunities for siring are high early in the season and diminish towards the end [17], while, in *P. alpina*, the advantage for female function of flowering later is probably due to the fact that it takes longer to produce more pistils and because flowers with more pistils in their centre experience a lower selfing rate (unpublished data). Importantly, we detected no sexual conflict over the number of stamens of bisexual flowers, and indeed stamen number was relatively uniform across flowers. It is also important to note that selection via the combined contributions to fitness of the two sexual functions was mostly driven by the male component, and indeed we detected no selection via the female function. This pattern of selection suggests that there is little selection that might favour the production of unisexual female flowers—in contrast to the strong selection favouring male flowers.

### (b) The early production of male flowers resolves sexual conflict

A key implication of our results is that the production of male flowers by *P. alpina* early in the flowering season at least partially resolves the sexual conflicts discussed above. Specifically, the distribution of pistil number and flowering date for male flowers (the two traits showing sexual conflict) were closer to their inferred optimal values. This finding illustrates how the modular growth and organization of plants allow them to produce flowers both with different genders and different associated traits, thereby breaking the constraint assumed in figure 1*b* that applies to traits measured at the level of individuals. In this sense, strategies such as monoecy or, here, andromonoecy allow functionally hermaphroditic plants to resolve conflict in a way similar to that enjoyed by dioecious, androdioecious or gynodioecious populations, in which males and females (or hermaphrodites) can express different traits [5,44,52].

The advantages of unisexuality as an effective resolution of sexual conflict faced by bisexual organisms may be responsible for evolutionary transitions toward sexual specialization and separate sexes [53]. The expression of gynodioecy in *Cyananthus delavayi* (in which individuals are either female or hermaphrodite) provides a revealing example. In *C. delavayi*, bisexual flowers are protandrous, and their pollen is presented to insect visitors on stylar hairs (a phenomenon known as secondary pollen presentation; Yeo [54]). Prolongation of the male function of bisexual flowers decreases the duration of their female function, greatly reducing the number of seeds produced. In *C. delavayi*, rather than resolving the conflict via the production of unisexual female flowers by otherwise hermaphroditic individuals (in analogy with what occurs in *P. alpina*), it appears that individuals have abandoned their male function altogether, rendering the population gynodioecious and allowing them to enjoy high reproductive success via their female function through the partial separation of the sexes in different individuals [48].

In contrast with the evolution of unisexuality at the individual level in gynodioecious *C. delavayi*, sexual conflict in *P. alpina* appears to have been resolved by separation of the sexes within individuals at a modular level, in this case via the expression of andromonoecy rather than androdioecy—the male counterpart of gynodioecy (conditions for the evolution of androdioecy are known to be more restrictive than those of gynodioecy [55,56]). It is possible that the mechanisms leading to the conflicts within bisexual flowers, i.e. a negative dependency of the selfing rate and onset of male function on female allocation, might not apply at the individual level in *P. alpina*. The reproductive success of well-resourced individuals of *P. alpina* with large numbers of male flowers is likely to be compromised by diminishing returns due to an increased selfing rate (but see figure S4 in electronic supplementary material) [57], pollen saturation on pollinators [58,59], or local mate competition [60,61].

In addition to their sex allocation and phenology, we found that male and bisexual flowers of *P. alpina* also differed in terms of the height of their subtending stalks (electronic supplementary material, table S5), with the floral stalks of bisexual flowers being much longer than those of male flowers. This result suggests that, here too, selection has been able to optimize a trait relatively independently for each of the two sexual functions. Our selection gradient analyses found no evidence for differences in selection on floral stalk height between the two sexes. This result may seem puzzling, yet is it likely that long floral stalks are particularly favourable to a plant's female function not during flowering but rather during fruiting and seed dispersal by wind [36,62]. Testing this hypothesis would require estimating the female component of fitness contributed by flowers not only in terms of the number of seeds they produce but also in terms of the distances over which their seeds are dispersed and the associated advantages of establishment and/or of avoiding the deleterious effects of kin competition [58,63,64].

### (c) Conflict resolution through divergence at individual versus modular levels of organization

Sexual conflict as a result of protogyny within flowers of *P. alpina* appears to have been at least partially resolved by divergence between male and female sex allocation, and by divergence between the sexes of phenological and morphological traits at the modular level. Interestingly, the suppression of female function in some flowers, thereby allowing flowers to commence their male function earlier and thus to profit from higher mating opportunities, has led to a protandrous sexual system at the individual level (with male flowers preceding bisexual flowers). This combination of protogyny at the flower level (i.e. in bisexual flowers) and protandry at the individual level (i.e. individuals producing both male and bisexual flowers) achieves something of the symmetry in mating opportunities that are realized in other species through the evolution of hermaphroditic dimorphisms such as heterodichogamy and flexistyly (see Introduction), where one class of individuals is protandrous and the other is protogynous [19,65].

Our study has focused on modular variation in sex allocation among flowers of *P. alpina*. The production of different male and bisexual flowers by an individual in the same reproductive season allows it to express different male and female (or hermaphrodite) traits, so that trait values are not constrained to be on the diagonal displayed in figure 1*b*. However, plants may achieve similar ends by separating their male and female functions in time, either within the same season or in different seasons. In *P. alpina*, small (and presumably young) individuals produce only male flowers and graduate as they become larger and older to producing hermaphrodite flowers [35]—a strategy known as ‘gender diphasy’ [66]. In *P. alpina* and several other species (e.g. [66–68]), such size-dependent gender switches can occur in both directions. The sequential hermaphroditism of many animals and gender diphasy in some plants provide revealing examples of how hermaphrodites may play the male and female roles at different times, allowing them to express different values of the same trait for each of their two sexual functions, e.g. size [69,70], colour and morphology [71], and behaviour [72,73].

### (d) The resolution of sexual conflict in hermaphrodite plants: concluding remarks

The high frequency of hermaphroditism in flowering plants strongly suggests that a bisexual strategy is often advantageous, despite the potential for conflict between the two sexual functions due to sexual interference within flowers. Although sexual interference has been considered an important reason for the evolution of a number of floral strategies and mating systems [5,15,57,74–78], it has rarely been viewed specifically in terms of intralocus sexual conflict ([79]; but see [48,80,81]). As our study demonstrates, sexual interference may be regarded as an example of sexual conflict in sex allocation traits, e.g. the number of female or male organs, where an increase of allocation to one sexual function decreases the reproductive success gained via the opposite function beyond a simple allocation trade-off, e.g. via increased selfing [82], ovule discounting [83] or reduced pollen export [16].

We suggest that sexual systems such as andromonoecy, gynomonoecy and monoecy (the expression of some male and/or female flowers by functionally hermaphroditic individuals) might often have evolved to resolve sexual conflict involving sex-allocation traits. Andromonoecy is probably a common evolutionary outcome of sexual conflict in bisexual flowers, with disruptive selection on pistil number via total reproductive success—favouring the production of flowers with high female allocation or no female allocation at all, as appears to be the case in *P. alpina.* In andromonoecious *Solanum carolinense*, longer pistils receive more outcross pollen (favouring female function) but interfere with pollen removal from the anthers when visited by bumblebees (compromising male function) [16]. In common with our results for *P. alpina*, there is probably no sexual conflict on allocation to stamens in *S. carolinense*, which attract and reward pollinators and thereby benefit both female and male functions [84]. In the case of gynomonoecy, a much rarer sexual system in which individuals produce both bisexual and female flowers [75,85,86], sexual conflict might cause disruptive selection on male allocation but not on female allocation, a scenario that mirrors our observations for andromonoecious *P. alpina*. Where sexual conflict causes disruptive selection on both female and male allocations, monoecy may represent an effective resolution [75]. The extent to which these sexual systems can be invoked as a general resolution of intra-floral sexual conflicts requires evaluation against other (non-mutually exclusive) explanations [75,87].

## Data Availability

All data underlying the analyses of this work are available on Dryad (https://doi.org/10.5061/dryad.rbnzs7hj0) [88]. Supplementary material is available online [89].
